# Characteristics of clinical trial websites: information distribution between ClinicalTrials.gov and 13 primary registries in the WHO registry network

**DOI:** 10.1186/1745-6215-15-428

**Published:** 2014-11-05

**Authors:** Daisuke Ogino, Kunihiko Takahashi, Hajime Sato

**Affiliations:** Department of Health Policy and Technology Assessment, National Institute of Public Health, 2-3-6 Minami, Wako, Saitama 351-0197 Japan; Department of Biostatistics, Nagoya University Graduate School of Medicine, Nagoya, Japan

**Keywords:** WHO International Clinical Trials Registry, Registry, Clinical trials, Website usability

## Abstract

**Background:**

It is well known that information about clinical trials is not easily accessible by the public. In Japan, clinical trial information can be accessed by the general public through online registries; however, many people find these registries difficult to use. To improve current clinical trial registries, we propose that combining them with clinical information phrased in lay terms would be beneficial to other interested professionals such as journalists and clinicians, as well as the general public. Therefore, this study aimed to examine the current pattern of distribution of clinical trial information from the primary World Health Organization (WHO) registries. Based on the results of this assessment, we then aimed to build and evaluate a prototype of the Japan Primary Registries Network (JPRN) portal that would be easily accessible to patients and the public, while still remaining useful for professionals.

**Methods:**

We assessed a total of 14 primary clinical trial registries listed on the WHO International Clinical Trials Registry Platform between January and February 2013. Website content was accessed and checked against a series of items that looked at usability, communication, design and accessibility of the sites. We excluded registries that were not active or were not on the approved WHO registry list at the time of our assessment. We also examined only the English versions of the websites as native-language registries may offer more functionality or different content than the English version of the same website.

**Results:**

All registries examined had a function allowing users to search the registry data and that displayed the related information from the search, including the clinical trial registration data. However, few websites were found to be user-friendly, and there was little integration with social media.

**Conclusions:**

We confirmed that there are few websites providing useful clinical trial information to patients and their families. However, information gleaned from some of the more advanced online registries could be used to improve the content and functionality of the JPRN portal.

## Background

The Japan Primary Registries Network (JPRN) is maintained by the Ministry of Health, Labour and Welfare in Japan (MHLW) and the National Institute of Public Health, Japan, which hosts the Clinical Trials Search portal [1]. The network comprises three primary registries: the University Hospital Medical Information Network [2]; the Japan Pharmaceutical Information Center’s Clinical Trials Information [3] and Japan Medical Association’s Center for Clinical Trials [4]. The JPRN portal was recognized as a World Health Organization (WHO) primary registry in 2008. It collects and manages clinical trial data in both Japanese and English. Several problems with the website have been pointed out by the clinical trial activation committee, including that patients found the portal difficult to use [5].

According to the 2012 Five-Year Clinical Trial Activation Plan proposed by the MHLW and the Ministry of Education, Culture, Sports, Science, and Technology, the goal is that patients and the public should be able to access the JPRN portal and learn from the clinical trial information stored within it, while researchers and clinicians can use the clinical trials information to help produce new innovations in Japanese medical treatment, such as new drugs [6]. Therefore, to improve the JPRN portal, we suggest that the current portal is combined with clear clinical information in lay terms to enhance the usability and understanding of clinical trials and other medical research (currently the portal does not focus exclusively on clinical trials, but also provides general medical information to the public) by the general public and any interested professionals [5, 6]. We propose that sharing the challenges of providing information between three data providers and the government would provide cohesive benefits for the JPRN. It would be possible to carry out joint improvement activities such as system maintenance, data formatting and quality control of the registration data. This could improve the system, including the website, while maintaining coordination with the network of the primary registry. Modification of the present Japanese clinical trial search portal site would address users’ requests for a more user-friendly and convenient website for all users, including patients and their families, medical professionals, pharmaceutical companies and researchers. Promoting participation in clinical trials and a greater understanding of clinical research would also be beneficial to the public [7]. Furthermore, an improvement in the quality of clinical trials, such as those investigating innovative new drugs, would be likely.

To determine the current pattern of distribution of clinical trial information from the WHO primary registries, an assessment needs to be conducted for each website. Based on the results of this assessment, a system prototype that could be easily accessed by patients and the general public and that would still be useful for medical professionals, could be built and evaluated against the goals proposed above.

## Methods

The websites of 14 clinical trial databases were assessed between January and February 2013. ClinicalTrials.gov (CT.gov) [8] and 13 primary registries listed in the WHO International Clinical Trials Registry Platform (ICTRP) [9] were included in this study (Table 1). We evaluated each registry against a checklist comprising 16 items related to website content (Table 2), 18 items on navigation, search and whether the website could be used in multiple languages (hereafter, referred to as multilingualization; Table 3), and 19 items related to website function, communication, design and accessibility (Table 4). All 14 websites were assessed by a web designer and developer. The checklist was prepared by all authors following a review of the literature. We focused on problems identified by the MHLW Clinical Trial Activation Committee [5] and the National Cancer Institute (NCI) International Clinical Trials Portal Website Usability Test Report [10].

**Table 1:** **List of websites assessed in this study**

Nation or region	Organization/URL
Japan	Japan Primary Registries Network (JPRN)
	http://rctportal.niph.go.jp/
USA	ClinicalTrials.gov (a service of the US National Institutes of Health)
	http://www.clinicaltrials.gov/
Australia and New Zealand	Australian New Zealand Clinical Trials Registry (ANZCTR)
	http://www.anzctr.org.au/
Brazil	Brazilian Clinical Trials Registry (ReBec)
	http://www.ensaiosclinicos.gov.br/
China	Chinese Clinical Trial Registry (ChiCTR)
	http://chictr.clinicaltrialecrf.org/en/
	(http://www.chictr.org/en/)
South Korea	Clinical Research Information Service (CRiS)
	http://ncrc.cdc.go.kr/cris/en/use_guide/cris_introduce.jsp
	(https://cris.nih.go.kr/cris/en/use_guide/cris_introduce.jsp)
Cuba	Cuban Public Registry of Clinical Trials (RPCEC)
	http://registroclinico.sld.cu/
EU	EU Clinical Trials Register (EU-CTR)
	https://www.clinicaltrialsregister.eu/
Germany	German Clinical Trials Register (DRKS)
	https://drks-neu.uniklinik-freiburg.de/drks_web/
Iran	Iranian Registry of Clinical Trials (IRCT)
	http://www.irct.ir/
UK	International Standard Randomised Controlled Trial Number Register (ISRCTN)
	http://www.isrctn.org/
Netherlands	Netherlands National Trial Register (NTR)
	http://www.trialregister.nl/trialreg/index.asp
Pan Africa	Pan African Clinical Trial Registry (PACTR)
	http://www.pactr.org/
Sri Lanka	Sri Lanka Clinical Trials Registry (SLCTR)
	http://www.slctr.lk/

**Table 2:** **Website content**

Assessment item	JPRN	CT.gov	ANZCTR	ReBec	ChiCTR	CRiS	RPCEC	EU CTR	DRKS	IRCT	ISRCTN	NTR	PACTR	SLCTR
Pages for each type of user, such as patients and health professionals	No	Partly	No	No	No	No	No	No	No	No	No	No	No	No
Content for each type of user, such as patients, health professionals and clients	No	Partly	No	No	No	No	No	No	No	No	No	No	No	No
Clinical trial information pages (patient)	–	–	–	–	–	–	–	–	–	–	–	–	–	–
Clinical trial information pages (health professional)	–	–	–	–	–	–	–	–	–	–	–	–	–	–
Information pages on diseases (patient)	No	No	No	No	No	No	No	No	No	No	No	No	No	No
Information pages on medicine and treatment for diseases (patient)	No	No	No	No	No	No	No	No	No	No	No	No	No	No
Links to information on diseases, drugs or treatments	No	No	No	No	No	No	No	No	No	No	No	No	No	No
List of ongoing clinical trial information	Yes	Yes	Yes	Yes	Yes	Yes	Yes	Yes	Yes	Yes	Yes	Yes	Yes	Yes
Information on the institutional review board (IRB)	No	Partly	No	No	No	No	No	No	Partly	No	No	No	No	Partly
IRB information page (patient)	–	Partly	No	No	No	No	No	No	No	No	No	No	No	No
IRB information page (health professional)	–	No	No	No	No	No	No	No	No	No	No	No	No	No
Use of videos and images	No	No	No	No	No	No	No	No	No	No	No	No	No	No
Frequently asked questions (patient)	Yes	Partly	No	No	Yes	No	No	Yes	Yes	Yes	No	No	Yes	Yes
Frequently asked questions (health professional)	–	–	Yes	Yes	No	No	No	–	–	–	Yes	No	–	–
Glossary (patient)	Yes	Yes	No	No	No	No	No	Yes	Yes	No	No	No	No	No
Glossary (health professional)	–	–	No	No	No	No	No	–	–	No	–	No	No	No

‘No’ encompasses ‘not provided’, ‘no description’ and ‘no distinction’. A dash (–) indicates that the response is summarized in the answer of another item or could not be evaluated.

**Table 3:** **Website multilingualization, navigation and search**

Assessment item	JPRN	CT.gov	ANZCTR	ReBec	ChiCTR	CRiS	RPCEC	EU CTR	DRKS	IRCT	ISRCTN	NTR	PACTR	SLCTR
**Multilingualization**														
Multilingualization of website	Yes	No	No	Yes	Yes	Yes	Yes	No	Yes	Yes	Yes	No	No	No
Multilingualization of contact information	Yes	–	–	No	Yes	Yes	Yes	–	Yes	Yes	No	Yes	No	No
Multilingualization of clinical trial information	Yes	–	–	Yes	Yes	Yes	Yes	–	Yes	Yes	No	No	No	No
**Navigation**														
Includes ‘clinical trial’ in the global navigation	No	Yes	Yes	Yes	Yes	No	No	No	No	No	No	No	No	No
Global navigation includes the roles ‘patient’ and ‘health professionals’	No	Partly	No	No	No	No	No	No	No	No	No	No	No	No
Minimum number of clicks to access clinical trial information (patient)	1	1	–	–	–	No	No	No	No	No	No	No	No	No
Minimum number of clicks to access clinical trial information (health professional)	–	1	–	–	–	No	No	No	No	No	No	No	No	No
Minimum number of clicks to access clinical trial information (practitioner)	–	1	–	–	–	No	No	No	No	No	No	No	No	No
Easy access to clinical trial information	Yes	Yes	–	–	–	No	–	Partly	–	–	Yes	–	–	–
Breadcrumb navigation	Yes	Yes	No	Yes	No	No	Yes	No	No	No	No	No	No	No
**Search**														
Search for clinical trial information	Yes	Yes	Yes	Yes	Yes	Yes	Yes	Yes	Yes	Yes	Yes	Yes	Yes	Yes
Optional search function for clinical trial information	Keywords, several conditions	Keywords, several conditions	Several conditions	Keywords, several conditions	Several conditions	Several conditions	Several conditions	Keywords, several conditions	Several conditions	Several conditions	Keywords	Several conditions	Keywords, several conditions	Several conditions
Display contents of search results	6 items	4 items	6 items	4 items	4 items	2 items	1 item	13 items	3 items	1 item	4 items	3 items	4 items	1 item
Overview display of search results for patients	No	No	Yes	No	No	No	No	No	No	Yes	No	No	No	No
Clinical trial search for each specific disease area	No	No	No	No	No	No	No	No	No	No	No	No	No	No
Information regarding past clinical trials	Yes	Yes	Yes	Yes	Yes	Yes	Yes	Yes	Yes	Yes	Yes	Yes	Yes	Yes
Information regarding clinical trials conducted in other facilities	Yes	Yes	Yes	Yes	Yes	Yes	Yes	Yes	Yes	Yes	Yes	Yes	Yes	Yes
Keyword shortcut to search	No	No	No	No	No	Yes	No	No	No	No	No	No	No	No

‘No’ encompasses ‘not provided’, ‘no description’ and ‘no distinction’. A dash (–) indicates that the response is summarized in the answer of another item or could not be evaluated.

**Table 4:** **Website function, communication, design and accessibility**

Assessment item	JPRN	CT.gov	ANZCTR	ReBec	ChiCTR	CRiS	RPCEC	EU CTR	DRKS	IRCT	ISRCTN	NTR	PACTR	SLCTR
**Function**														
Clinical trial data output (RSS, XML or CSV)	No	Yes	Yes	Yes	No	Yes	No	Yes	Yes	No	No	No	No	No
Function to evaluate the content	No	No	No	No	No	No	No	No	No	No	No	No	No	No
Function to submit opinions and requests	Partly	Yes	Yes	No	No	No	No	Yes	Yes	No	Partly	No	Yes	No
**Communication**														
Use of social media	No	No	No	No	No	No	No	No	No	No	Yes	No	No	No
Email address	Partly	No	Yes	Yes	Yes	No	Yes	Yes	Yes	Yes	Yes	Yes	Yes	Yes
Phone number	Yes	No	Yes	Yes	Yes	Yes	Yes	No	Yes	Yes	Yes	No	Yes	Yes
Fax number	Yes	No	Yes	No	Yes	Yes	No	No	Yes	Yes	Yes	No	Yes	Yes
Transportation guide	Yes	No	No	No	No	–	No	No	No	No	No	No	No	No
Inquiry form	Yes	Yes	Yes	Yes	No	–	Yes	No	Yes	Yes	No	No	Yes	No
Contact for each purpose	Yes	No	No	No	No	–	No	No	No	No	No	No	No	No
Alternate contact method	No	No	No	No	No	No	No	No	No	No	No	No	No	No
How to apply for the clinical trial	No	Yes	No	No	No	No	No	No	No	No	No	No	No	No
Apply for the clinical trial from the website	No	No	No	No	No	No	No	No	No	No	No	No	No	No
**Design**														
Website compatible with smartphones	No	No	No	No	No	No	No	No	No	No	No	No	No	No
Website compatible with feature phones	No	No	No	No	No	No	No	No	No	No	No	No	No	No
Website compatible with tablets	No	No	No	No	No	No	No	No	No	No	No	No	No	No
**Accessibility**														
Font size	No	No	No	No	No	No	Yes	No	Yes	No	No	No	Yes	No
Images with alt attribute	Yes	Yes	Yes	Yes	Partly	Partly	Yes	Yes	Yes	No	No	Partly	Partly	No
Elimination of layout by < table > tag	Yes	Yes	Yes	Yes	Yes	Yes	Yes	No	Yes	No	No	No	Yes	No

‘No’ encompasses ‘not provided’, ‘no description’ and ‘no distinction’. A dash (–) indicates that the response is summarized in the answer of another item or could not be evaluated.

CSV, comma-separated values; RSS, RDF site summary; XML, extensible markup language.

The assessment results were analyzed to determine patterns in how clinical trial information was provided. We assessed only the English versions of the included registries (with the exception of the JPRN portal site, for which the Japanese version was assessed), as native-language versions of these websites may offer more functions or different content than the English versions. With respect to institutional review board (IRB) information, because we only searched the English websites of each registry, it is possible that information was missed if it was included using terms other than ‘IRB’ or was written in the authors’ native language. The IRB information may also contain information that overlaps with other ethics committee information. This was a limitation here, in that we were not able to check in the native language of the registry whether the item had been provided. We excluded registries that were either not active or that were not on the WHO list of approved registries at the time of our assessment. We did not correspond with the administrators of any of the registries to clarify, supplement or verify the information extracted. The assessment was completed based on the information provided on each of the 14 registry websites.

## Results

### Website content

The CT.gov website contained content and parts of pages devoted to each type of user (laypeople and professionals such as clinicians, funders, journal editors, journalists, systematic reviewers and researchers), although the distinction was not always clear. None of the 14 websites had separate pages with information on clinical trial information, disease information, medicine and treatments for diseases, or links to similar information. All 14 websites had a list of ongoing clinical trial data. Eleven websites incorporated frequently asked questions, and four websites included a glossary (Table 2).

### Website multilingualization, navigation and search

Regarding the use of multiple languages on the websites, organizations with a native language other than English were presumed to use multiple languages. Global navigation was one of the usability items assessed. Global navigation systems place page links onto the website homepage, and function as a shortcut to the content of the website. Global navigation can help users understand the complete scope of the website or their current position in the website. Breadcrumb navigation displays web pages within a hierarchy on the website. This display makes it easy to determine where the user is located within the website and allows users to jump to other pages in the hierarchy via links, making the website easier to navigate. We assessed each website in terms of what was displayed in the banner area on the homepage and the global navigation system by which users can move around the site. Four websites included ‘clinical trial’ in their global navigation options, four websites facilitated easy access to clinical trial information, and four websites had breadcrumb navigation features. The definition of ‘easy access’ followed NCI recommendation 7 in the Website Usability Test Report [10]: ‘Redesign the home page to reduce the amount of text and make links to important topics on third-level pages visible’.

As CT.gov was the only website to offer a separate page for each user type, it was also the only registry to which certain items applied: ‘Global navigation includes the roles “patient” and “health professionals”’, and ‘Minimum number of clicks to access clinical trial information’.

All registry websites had a search function for clinical trial information and displayed information on past clinical trials and trials conducted in other facilities. A few websites had functions such as an overview display of search results for patients, and keyword shortcuts for searching (Table 3).

### Website function, communication, design and accessibility

#### Website function and communication

Six websites had an output function for clinical trial data: data could be retrieved in RSS (RDF site summary), XML (extensible markup language) or CSV (comma-separated values) format. Seven websites had a function for users to submit opinions and requests. However, no website had a function to allow users to evaluate the website content. The International Standard Randomised Controlled Trial Number Register (ISRCTN.org) site [11] was the only website that used social media (Twitter).

#### Website design and accessibility

None of the websites were designed to be natively compatible with smartphones, feature phones or tablets. Three websites included features to adjust the font size. The aim of the JPRN portal site is to be accessible to all people, including disabled people. The following items were included in the category for accessibility.

Alt attributes, cascading style sheets (CSS) and tableless design are relevant for accessibility. The alt attribute is the alternative text attached to an image. For users who need a voice browser, it is the string that can be read by a text-to-speech browser and that is displayed when the image is not found. Six websites attached an alt attribute almost perfectly; however, images were not widely used on three websites. Use of a CSS layout (used instead of a table layout) along with the < table > tag to present data as a table, avoids the use of tables as a basis for the entire webpage layout (which was a feature of earlier web browsers). Without this feature, for users who need a voice browser, it is not possible to distribute the contents accurately because the structure of the content is dependent on appearance. The layouts of seven websites used CSS instead of the table layout using the < table > tag (Table 4).

## Discussion

The results of this assessment demonstrate that few websites (particularly those in a native language other than English) were easily accessible by patients and the general public for locating information on clinical trials. Currently, the NCI [12] website allows users to search for clinical cancer trials using data imported from CT.gov. Before CT.gov began to administer the website, NCI had its own original database of clinical trials from the 1970s. At that time, there were two versions of trial descriptions, one for health professionals and one for patients. The patient version displayed optional information on the purpose, eligibility, treatment/intervention, lead organizations, trial sites and contacts. Moreover, the current NCI website is a very good example of efforts to create and improve a website for patients and the general public. Dear *et al*. pointed out that the most important additional item for website users was the lay summary [13, 14]. With an overview display of clinical trial search results, patients and the public can easily understand the contents and whether particular clinical trials are associated with their disease. In addition, a lay summary of the information may help in their decision-making regarding which clinical trials to join [15].

Several problems with clinical trial registry websites that we encountered were identified by the Clinical Trial Activation Committee [5]. One issue is that people cannot find information on the clinical research or trials related to their own diseases, e.g., how many clinical trials are being conducted or if they are taking place at a nearby location. It is also a problem that the total number of clinical trials that have been conducted in Japan cannot be calculated (some clinical trials conducted in Japan were registered in overseas registries). Grobler *et al*. pointed out the problem of duplicate trial registration [15, 16]. Some organizations have already identified this as an important issue, and several (e.g. CT.gov and ICTRP) have made efforts to reduce duplicate registrations by using unique identifiers. To avoid publication bias and selective reporting, the WHO Trial Registration Data Set comprising 20 data elements has been approved by the International Committee of Medical Journal Editors [9, 17, 18]. However, only a few items pertain to those patients and individuals in the general population who are asking for specific and prioritized information. Some of the requested data elements may be unavailable to them. Further investigation is needed to determine which information is most desired by patients and the general public.

We have not yet had much opportunity to ascertain user opinions about the JPRN portal. Despite limited personnel and budget, there is a need to continuously improve the website and to evaluate the site contents by methods such as satisfaction surveys. CT.gov and NCI conduct a standard survey to address this need: the American Customer Satisfaction Index Online Consumer Survey [19]; however, other surveys regarding website usability and internet user satisfaction were not found in this study. To improve a website, at the very least, current information is required on how users access the website and use the search function. Access log analysis is one way to understand the behavior of internet information seekers [20, 21]. Another option is the use of website evaluations that are accessible and user-friendly and therefore provide a good opportunity for receiving feedback directly from users. However, feedback should be verified against the content provided and the actual information requested by users [22–24]. Improved accessibility of websites is likely to make a good impression on all users.

CT.gov's clinical trials database is the largest of all the organizations assessed (excluding ICTRP), and it has a long history of system operation and system management [25]. Clinical trial registration is required by federal law in the United States [26–29] and by the European Medicines Agency [30] in the EU. However, in Japan, there is currently no such legislation. If such legislation were introduced, it might be possible for the JPRN to create a common format or dataset, and the addition of common data elements for clinical trial registration (excluding the 20 minimum data elements proposed by WHO [31]) could proceed and be harmonized smoothly. In addition, the current JPRN website does not offer a means for clinical trial data output. This limits distribution to the public, except via the ICTRP, because there is no authentication of the data provided by the three organizations. In addition, there are a few unique problems with the JPRN portal site: it is not linked to an adequate number of hospitals or associations; few of the general public know of its existence or how to access it; and patients and the public are not aware of the useful information it contains [32, 33]. It is possible that the National Institute of Public Health will begin to play a role in the management and distribution of clinical research and clinical trial information in Japan. There are still many challenges for organizations to overcome, including the distribution of clinical trial information to patients and their families [15, 34, 35]. A big challenge is the format of the data in JPRN: a different Japanese format and system are used by each of the three organizations. Therefore, it is necessary to reform the legal system with respect to new data input into the portal; it should be mandated that lay terms be provided at registration to enable patients and the general public to understand them more easily.

## Conclusions

In this study, the content and the characteristics of the online registry systems were found to be different for each organization. We confirmed that there were few websites that provided useful clinical trial information to patients and the general public. It is likely that the number of years of website operation, the amount of integrated data collected on clinical trials, and regional characteristics and resources (personnel and budget) differ by organization and thus affect their content and design. Moreover, concerning the dissemination of their services to patients and the public, we discovered room for improvement in the JPRN content and website systems. It follows that there may be other organizations that have similar challenges.

The distribution of clinical trial information to interested parties continues to be a challenge for WHO primary registries. Recommendations to improve the content and functions of these registries (including the JPRN) can be made based on advanced versions of other current websites, specifically, CT.gov and the NCI website.

## Abbreviations

ANZCTR: Australian New Zealand Clinical Trials Registry
ChiCTR: Chinese Clinical Trial Registry
CSS: cascading style sheets
CRiS: Clinical Research Information Service
CT.gov: ClinicalTrials.gov
DRKS: German Clinical Trials Register
EU-CTR: EU Clinical Trials Register
ICTRP: International Clinical Trials Registry Platform
IRB: institutional review board
IRCT: Iranian Registry of Clinical Trials
ISRCTN: International Standard Randomised Controlled Trial Number Register
JPRN: Japan Primary Registries Network
MHLW: Ministry of Health, Labour and Welfare
NCI: National Cancer Institute
NTR: Netherlands National Trial Register
PACTR: Pan African Clinical Trial Registry
ReBec: Brazilian Clinical Trials Registry
RPCEC: Cuban Public Registry of Clinical Trials
SLCTR: Sri Lanka Clinical Trials Registry
WHO: World Health Organization.

## References

[1] **National Institute of Public Health, Clinical Trials Search** [http://rctportal.niph.go.jp/en/index]

[2] **University Hospital Medical Information Network (UMIN)** [http://www.umin.ac.jp/ctr/]

[3] **Japan Pharmaceutical Information Center, Clinical Trials Information (JapicCTI)** [http://www.japic.or.jp/]

[4] **Japan Medical Association, Center for Clinical Trials (JMACCT)** [http://www.jmacct.med.or.jp/en/]

[5] *Proceedings of the Fourth Clinical Research/Trial Activation Study Group in the Ministry of Health, Labour and Welfare: 2011 December 7 (in Japanese)*. [http://www.mhlw.go.jp/stf/shingi/2r9852000001zf1q.html]

[6] Ministry of Health, Labour and Welfare: **Clinical research/trial activation five-year plan. 2012 (in Japanese).** [http://www.mhlw.go.jp/topics/bukyoku/isei/chiken/dl/121025_3.pdf]

[7] The Association of the British Pharmaceutical Industry (2008). Best practice model for the disclosure of results and transparent information on clinical trials.

[8] **US National Institutes of Health, ClinicalTrials.gov** [http://clinicaltrials.gov/]

[9] **The World Health Organization, international clinical trials registry platform (ICTRP)** [http://www.who.int/ictrp/en/]

[10] National Cancer Institute (2011). International Clinical Trials Portal Website Usability Test: Report of Findings and Recommendations.

[11] **Current Controlled Trials, International Standard Randomised Controlled Trial Number Register (ISRCTN)** [http://www.controlled-trials.com/]

[12] **US National Institutes of Health, National Cancer Institute (NCI)** [http://www.cancer.gov/]

[13] Dear R, Barratt A, Askie L, McGeechan K, Arora S, Crossing S, Currow D, Tattersall M (2011). Adding value to clinical trial registries: insights from Australian Cancer Trials Online, a website for consumers. Clin Trials.

[14] Dear RF, Barratt AL, Crossing S, Butow PN, Hanson S, Tattersall MH (2011). Consumer input into research: the Australian Cancer Trials website. Health Res Policy Syst.

[15] Faure H, Hrynaszkiewicz I (2011). The ISRCTN Register: achievements and challenges 8 years on. J Evid Based Med.

[16] Grobler L, Siegfried N, Askie L, Hooft L, Tharyan P, Antes G (2008). National and multinational prospective trial registers. Lancet.

[17] DeAngelis CD, Drazen JM, Frizelle FA, Haug C, Hoey J, Horton R, Kotzin S, Laine C, Marusic A, Overbeke AJ, Schroeder TV, Sox HC, Van Der Weyden MB, International Committee of Medical Journal Editors (2004). Clinical trial registration: a statement from the International Committee of Medical Journal Editors. JAMA.

[18] Askie L, Ghersi D, Simes J (2006). Prospective registration of clinical trials. Aust J Physiother.

[19] **The American Customer Satisfaction Index (ACSI)** [http://www.theacsi.org/the-american-customer-satisfaction-index]

[20] Wallwiener M, Wallwiener CW, Brucker SY, Hartkopf AD, Fehm TN, Kansy JK (2010). The Brustkrebs-Studien.de website for breast cancer patients: user acceptance of a German internet portal offering information on the disease and treatment options, and a clinical trials matching service. BMC Cancer.

[21] Patel CO, Garg V, Khan SA (2010). What do patients search for when seeking clinical trial information online?. AMIA Annu Symp Proc.

[22] Eysenbach G, Köhler C (2002). How do consumers search for and appraise health information on the world wide web? Qualitative study using focus groups, usability tests, and in-depth interviews. BMJ.

[23] Grama LM, Beckwith M, Bittinger W, Blais D, Lollar C, Middleswarth A, Noone M, Price D, Quint-Kasner S, Shields V, Wright LW (2005). The role of user input in shaping online information from the National Cancer Institute. J Med Internet Res.

[24] Peterson G, Aslani P, Williams KA (2003). How do consumers search for and appraise information on medicines on the Internet? A qualitative study using focus groups. J Med Internet Res.

[25] Califf RM, Zarin DA, Kramer JM, Sherman RE, Aberle LH, Tasneem A (2012). Characteristics of clinical trials registered in ClinicalTrials.gov, 2007–2010. JAMA.

[26] Zarin DA, Keselman A (2007). Registering a clinical trial in ClinicalTrials.gov. Chest.

[27] Tse T, Williams RJ, Zarin DA (2009). Reporting ‘basic results’ in ClinicalTrials.gov. Chest.

[28] Tse T, Williams RJ, Zarin DA (2009). Update on registration of clinical trials in ClinicalTrials.gov. Chest.

[29] Zarin DA, Tse T, Williams RJ, Califf RM, Ide NC (2011). The ClinicalTrials.gov results database – update and key issues. N Engl J Med.

[30] **European Medicines Agency, EU Clinical Trials Register** [https://www.clinicaltrialsregister.eu/]

[31] **WHO Trial Registration Data Set (Version 1.2.1)** [http://www.who.int/ictrp/network/trds/en/]

[32] McCabe S (2005). Open access to trials register. PLoS Med.

[33] Mosconi P, Roberto A (2012). Open-access clinical trial registries: the Italian scenario. Trials.

[34] Askie LM (2011). Australian New Zealand Clinical Trials Registry: history and growth. J Evid Based Med.

[35] Hasselblatt H, Dreier G, Antes G, Schumacher M (2009). The German Clinical Trials Register: challenges and chances of implementing a bilingual registry. J Evid Based Med.

